# A Deubiquitylating Complex Required for Neosynthesis of a Yeast Mitochondrial ATP Synthase Subunit

**DOI:** 10.1371/journal.pone.0038071

**Published:** 2012-06-19

**Authors:** Sophie Kanga, Delphine Bernard, Anne-Marie Mager-Heckel, Zoi Erpapazoglou, Francesca Mattiroli, Titia K. Sixma, Sébastien Léon, Danièle Urban-Grimal, Ivan Tarassov, Rosine Haguenauer-Tsapis

**Affiliations:** 1 Institut Jacques Monod, CNRS, UMR7592, Univ Paris Diderot, Sorbonne Paris Cité, Paris, France; 2 Division of Biochemistry, Netherlands Cancer Institute, Amsterdam, The Netherlands; 3 UMR 7156 Génétique Moléculaire, Génomique, Microbiologie (GMGM), Université de Strasbourg - CNRS, Strasbourg, France; Karolinska Institutet, Sweden

## Abstract

The ubiquitin system is known to be involved in maintaining the integrity of mitochondria, but little is known about the role of deubiquitylating (DUB) enzymes in such functions. Budding yeast cells deleted for *UBP13* and its close homolog *UBP9* displayed a high incidence of *petite* colonies and slow respiratory growth at 37°C. Both Ubp9 and Ubp13 interacted directly with Duf1 (DUB-associated factor 1), a WD40 motif-containing protein. Duf1 activates the DUB activity of recombinant Ubp9 and Ubp13 *in vitro* and deletion of *DUF1* resulted in the same respiratory phenotype as the deletion of both *UBP9* and *UBP13.* We show that the mitochondrial defects of these mutants resulted from a strong decrease at 37°C in the *de novo* biosynthesis of Atp9, a membrane-bound component of ATP synthase encoded by mitochondrial DNA. The defect appears at the level of *ATP9* mRNA translation, while its maturation remained unchanged in the mutants. This study describes a new role of the ubiquitin system in mitochondrial biogenesis.

## Introduction

Ubiquitylation is a posttranslational modification in which ubiquitin, a highly conserved 76-residue polypeptide, is attached to target proteins through a series of enzymatic reactions involving a ubiquitin-activating enzyme (E1), ubiquitin-conjugating enzymes (E2), and ubiquitin protein ligases (E3) involved in substrate recognition. The best known of the many functions of ubiquitin is the targeting of proteins for degradation by the proteasome. However, ubiquitylation can also signal non proteolytic functions in many cellular processes including the cell cycle, gene expression, and protein trafficking [1]. Ubiquitylation is a highly versatile means of regulating protein function, activity, stability, distribution and interactions in the cell.

The mechanisms of ubiquitin conjugation have been studied extensively, but far less is known about the removal of ubiquitin by deubiquitylating enzymes (DUBs). Deubiquitylation is required: (i) to cleave ubiquitin from its precursors, (ii) to maintain a pool of free ubiquitin in the cell, (iii) to antagonize ubiquitylation of substrates. Nineteen putative DUBs have been identified in yeast [2], [3] and about 95 have been identified in human [4]. Most of the yeast DUBs belong to the UBP (ubiquitin-specific protease) subfamily. These proteins are cysteine proteases containing two well conserved protein sequences, the Cys and His boxes. These domains contain the catalytic triad residues and other residues of the active site pocket. Despite extensive functional analysis, phenotypic defects were found in only a few of the single *ubp* mutants. Moreover, the regulation of DUB activity and substrate specificity remains poorly understood, although most DUBs are found associated with other proteins, suggesting that partner proteins may play a regulatory role [4].

The ubiquitin system has been found to influence mitochondrial functions in many ways, in both yeast and mammals (reviewed in [5]). It is required for functions as diverse as protein import [6], [7], tRNA import [8], transport to mitochondria of phosphatidyl serine synthesized in the endoplasmic reticulum (ER) [9], stability of mitochondrial DNA (mtDNA) [10] and mtDNA segregation [11]. The ubiquitin proteasome system also appears to be required for various aspects of mitochondrial quality control processes; notably under conditions of mitochondrial stress, for the degradation of proteins of the outer mitochondrial membrane [12], and even for the degradation of intramitochondrial proteins, in a process that, like endoplasmic reticulum-associated degradation (ERAD), may involve retrotranslocation from the mitochondria to the cytoplasm before ubiquitylation and proteasomal degradation [13], [14], [15]. The most documented function of the ubiquitin system in mitochondria is probably its role in mitochondrial morphology and dynamics. Several E3 enzymes, either cytoplasmic or associated with the mitochondrial outer membrane, are required for mitochondrial fusion-fission processes. The yeast cytosolic F-box protein Mdm30 controls the turnover of the mitofusin Fzo1 required for mitochondrial fusion [16], in an ubiquitin-dependent manner [17]. In mammalian cells, recent studies have identified two E3s embedded in the mitochondrial outer membrane, that are involved in mitochondrial dynamics [18], [19]. Currently, little is known about the role of DUBs in mitochondrial functions. In budding yeast, Ubp16 was the only UBP known to be associated with mitochondria [20]. No mitochondrial function has yet been demonstrated for this DUB, but its putative ortholog in mammals, USP30, is thought to be involved in regulating mitochondrial morphology [21].

To identify UBPs required for normal mitochondrial function, we investigated the respiratory growth and the incidence of *petite* colonies for each *ubp* deletion mutant in the yeast *Saccharomyces cerevisiae.* We found that two homologous DUBs, Ubp9 and Ubp13, have redundant roles in mitochondrial function. We identified a new WD40 protein that interacts with these two DUBs, that we named Duf1 (DUB-associated factor 1), and which is also required for mitochondrial function. We then investigated the mitochondrial process involving these two DUBs and their partner. Our data led us to focus on the mitochondrial ATP synthase whose primary function is to use the electrochemical gradient generated by the respiratory chain to produce ATP from ADP and inorganic phosphate [22]. ATP synthase consists of the hydrophilic F1 catalytic moiety and a hydrophobic moiety, F0, located in the inner mitochondrial membrane. Our data show that Ubp9, Ubp13 and Duf1 are involved in the biosynthesis of the mitochondrial-encoded Atp9, an essential subunit of the hydrophobic F0 moiety of the ATP synthase, providing a new link between the ubiquitin system and mitochondrial function through the biogenesis of the F0 complex.

## Results

### Deletion of Both *UBP9* and *UBP13,* or of *DUF1* Alone, Impair Mitochondrial Function

**Figure 1:**
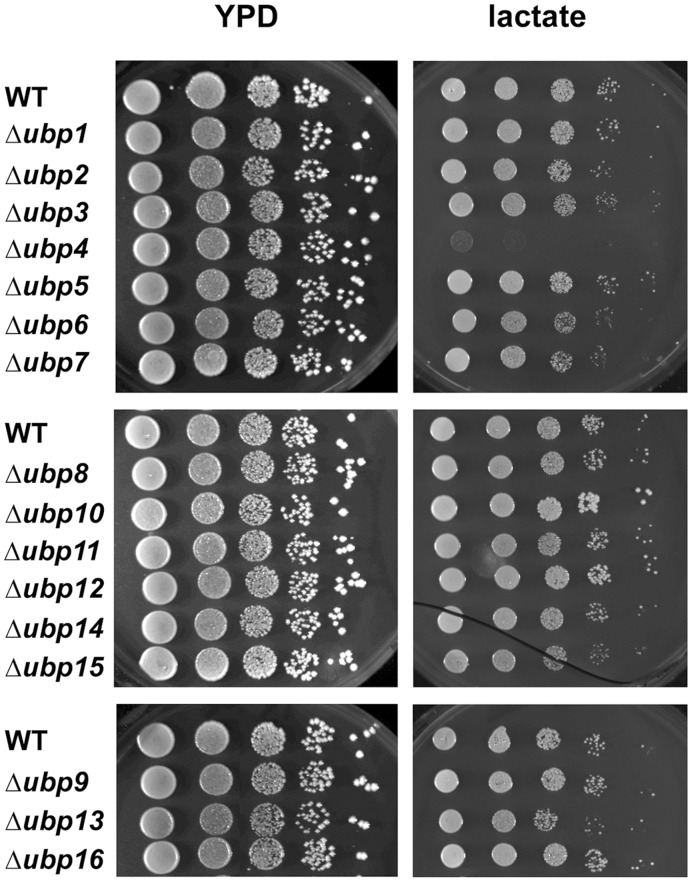
Respiratory growth of yeast *ubp* mutants. Dilution series of wild-type BY4741 (WT) and Δ*ubpx* strains were grown on media containing fermentable (glucose) or respiratory (lactate) substrates, at 30°C, for 3 and 5 days, respectively. Similar results were obtained with ethanol and glycerol as respiratory substrates.

We used two different approaches to investigate the possible involvement of yeast UBPs in mitochondrial function. We first assessed the respiratory growth of all of the single *UBP* deletion mutants (listed Table 1) on media containing non fermentable carbon sources lactate (Fig. 1) or ethanol/glycerol (data not shown) that can only be metabolized by oxidative phosphorylation. We also checked the incidence of *petite* colonies (Table 2), a phenotype corresponding to extensive deletions (rho^−^) or a complete absence of mitochondrial DNA (rho^0^) [23]. Only Δ*ubp4* (Δ*doa4*) cells, which have low ubiquitin levels (supplemental Fig. S1 and [24]) and defects in multiple ubiquitin-dependent processes [25], [26], displayed a major growth defect on lactate medium (Fig. 1). Consistent with the respiratory growth phenotype, we found that Δ*ubp4* cells displayed a high incidence of *petite* colonies. A high frequency of *petite* colonies was also observed for Δ*ubp6,* Δ*ubp8,* Δ*ubp13* and Δ*ubp15* cells (Table 2). The half-life of ubiquitin is known to be short in Δ*ubp6* cells [27], resulting in defects in a number of ubiquitin-dependent processes. Ubp8 is involved in the deubiquitylation of histone H2B and, thus, in the transcriptional regulation of multiple genes [28], which may indirectly affect mitochondrial function. Δ*ubp13* and Δ*ubp15* mutants have never been reported to display altered mitochondrial function. We decided to focus here on Ubp13.

**Table 1:** Genotypes and sources of yeast strains.

Strain	Genotype	Source
BY4741	Mat a *his3*Δ*1 leu2*Δ*0 met15*Δ*0 ura3*Δ*0*	Euroscarf
BY4742	Mat α *his3*Δ*1 leu2*Δ*0 lys2*Δ*0 ura3*Δ*0*	Euroscarf
YDB105	Mat α *UBP9-HA_3_-HIS3MX6 his3*Δ*1 leu2*Δ*0 lys2*Δ*0 ura3*Δ*0*	This study
YDB106	Mat α *UBP13-HA_3_-HIS3MX6 his3*Δ*1 leu2*Δ*0 lys2*Δ*0 ura3*Δ*0*	This study
YDB107	Mat α *DUF1-HA_3_-HIS3MX6 his3*Δ*1 leu2*Δ*0 lys2*Δ*0 ura3*Δ*0*	This study
DB122-1D	Mat a *UBP9-GFP-HIS3MX6 UBP13-HA_3_-HIS3MX6 his3*Δ*1 lys2*Δ*0 ura3*Δ*0*	This study
DB126-1A	Mat a *DUF1-GFP-HIS3MX6 UBP13-HA_3_-HIS3MX6 his3*Δ*1 leu2*Δ*0 ura3*Δ*0*	This study
Δ*duf1 a*	Mat a *duf1::kanMX4 his3*Δ*1 leu2*Δ*0 met15*Δ*0 ura3*Δ*0*	Euroscarf
Δ*duf1* α	Mat α *duf1::kanMX4 his3*Δ*1 leu2*Δ*0 lys2*Δ*0 ura3*Δ*0*	Euroscarf
YDB104	Mat a *ubp9::kanMX4 ubp13::HIS3MX6 his3*Δ*1 leu2*Δ*0 met15*Δ*0 ura3*Δ*0*	This study
DB108-8C	Mat a *ubp9::kanMX4 ubp13::HIS3MX6 yol087c::kanMX4 his3*Δ*1 leu2*Δ*0 met15*Δ*0 ura3*Δ*0*	This study
DB109-5B	Mat α *ubp9::kanMX4 ubp16::kanMX4 his3*Δ*1 leu2*Δ*0 lys2*Δ*0 ura3*Δ*0*	This study
DB109-8D	Mat α *ubp9::kanMX4 ubp16::kanMX4 his3*Δ*1 leu2*Δ*0 lys2*Δ*0 ura3*Δ*0*	This study
DB109-11C	Mat α *ubp13::HIS3MX6 ubp16::kanMX4 his3*Δ*1 leu2*Δ*0 lys2*Δ*0 ura3*Δ*0*	This study
DB109-13C	Mat a *ubp13::HIS3MX6 ubp16::kanMX4 his3*Δ*1 leu2*Δ*0 met15*Δ*0 ura3*Δ*0*	This study
DB109-5C	Mat a *ubp9::kanMX4 ubp13::HIS3MX6 ubp16::kanMX4 his3*Δ*1 leu2*Δ*0 met15*Δ*0 ura3*Δ*0*	This study
DB109-8B	Mat a *ubp9::kanMX4 ubp13::HIS3MX6 ubp16::kanMX4 his3*Δ*1 leu2*Δ*0 met15*Δ*0 ura3*Δ*0*	This study
YDB123	Mat a *DUF1-HA_3_-HIS3 ura3-1 leu2-3,112 his3-115*	This study
YDB124	Mat a *DUF1-HA_3_-HIS3 pre1-1 pre2-2 ura3-1 leu2-3,112 his3-115*	This study
DB127-3D	Mat a *UBP9-HA_3_-HIS3 duf1::kanMX4 his3*Δ*1 leu2*Δ*0 met15*Δ*0 ura3*Δ*0*	This study
DB128-2C	Mat a *UBP13-HA_3_-HIS3 duf1::kanMX4 his3*Δ*1 leu2*Δ*0 met15*Δ*0 ura3*Δ*0*	This study
DB133-8C	Mat a *DUF1-HA_3_-HIS3 ubp9::kanMX4 ubp13::HIS3MX6 his3*Δ*1 leu2*Δ*0 met15*Δ*0 ura3*Δ*0*	This study
DB133-8D	Mat α *DUF1-HA_3_-HIS3 ubp13::HIS3MX6 his3*Δ*1 leu2*Δ*0 lys2*Δ*0 ura3*Δ*0*	This study
DB133-9C	Mat a *DUF1-HA_3_-HIS3 ubp9::kanMX4 his3*Δ*1 leu2*Δ*0 met15*Δ*0 ura3*Δ*0*	This study
Δ*ubpn* ^(1)^	Mat a *ubpn::kanMX4 his3*Δ*1 leu2*Δ*0 met15*Δ*0 ura3*Δ*0*	Euroscarf

Δ*ubpn* refers to a collection of strains, each with the deletion of a single *UBP* gene (1≤ n ≤16).

**Table 2:** Incidence of petite colonies in Δ*ubpn* cells.

Main genetic background	% *petite* colonies	Number of colonies counted
wild type BY	1,8%	983
Δ*ubp1*	1,4%	501
Δ*ubp2*	5,8%	361
Δ*ubp3*	0,5%	407
Δ*ubp4*	38.5%	1024
Δ*ubp5*	2.2%	402
Δ*ubp6*	15.8%	487
Δ*ubp7*	2.3%	655
Δ*ubp8*	23.6%	828
Δ*ubp9*	7.9%	291
Δ*ubp10* *	0.5%	407
Δ*ubp11*	7.8%	344
Δ*ubp12*	2.0%	596
Δ*ubp13*	20.0%	364
Δ*ubp14*	2.3%	210
Δ*ubp15*	23.0%	496
Δ*ubp16*	3.6%	448

* DF5 Genetic background.

For each strain, one respiratory competent colony was streaked on glucose medium. After 3 days of incubation at 30°C, the resulting colonies, of both small and regular size, were counted.

**Figure 2:**
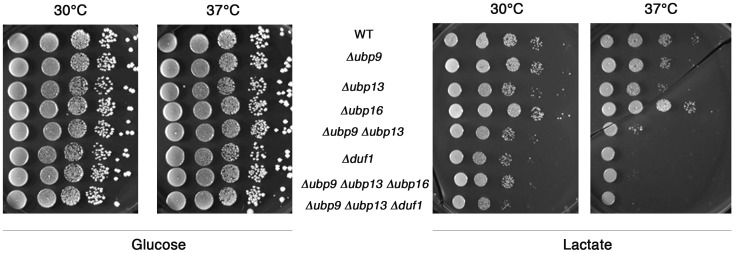
Δ*ubp9* Δ*ubp13* and Δ*duf1* mutants have a similar respiratory phenotype, which is not aggravated by the deletion of *UBP16*. Dilution series of wild-type BY4741 (WT), Δ*ubp9*, Δ*ubp13,* Δ*ubp16,* Δ*ubp9* Δ*ubp13*, Δ*duf1,* Δ*ubp9* Δ*ubp13* Δ*ubp16* and Δ*ubp9* Δ*ubp13* Δ*duf1* strains were grown on medium containing fermentable (glucose) or respiratory (lactate) substrates for 5 days at 30°C and 37°C.

As the sequence of Ubp13 is 45% identical to that of Ubp9, along its entire length [2], we analyzed in more detail the respiratory growth of cells harboring a single or double deletion of *UBP9* and *UBP13.* Experiments were performed at two different temperatures, because mutants displaying a thermosensitive mitochondrial phenotype have been described [29], [30]. Single deletions of *UBP9* or *UBP13* resulted in no major growth defect under these conditions, although a slight defect was observed for Δ*ubp13* at 37°C. By contrast, the Δ*ubp9* Δ*ubp13* double mutant grew slower on lactate (Fig. 2) and ethanol/glycerol (data not shown) media at 30°C, and displayed a strong growth defect at 37°C (Fig. 2), indicating a possible redundancy in the function of these two proteins. The deletion of *UBP16,* which encodes the only yeast mitochondrial Ubp identified to date, did not lead to any defective respiratory phenotype [20], and did not further aggravate the respiratory deficiency observed in the Δ*ubp9* Δ*ubp13* double mutant (Fig. 2), suggesting that Ubp16 is not redundant with Ubp9 and Ubp13.

**Figure 3:**
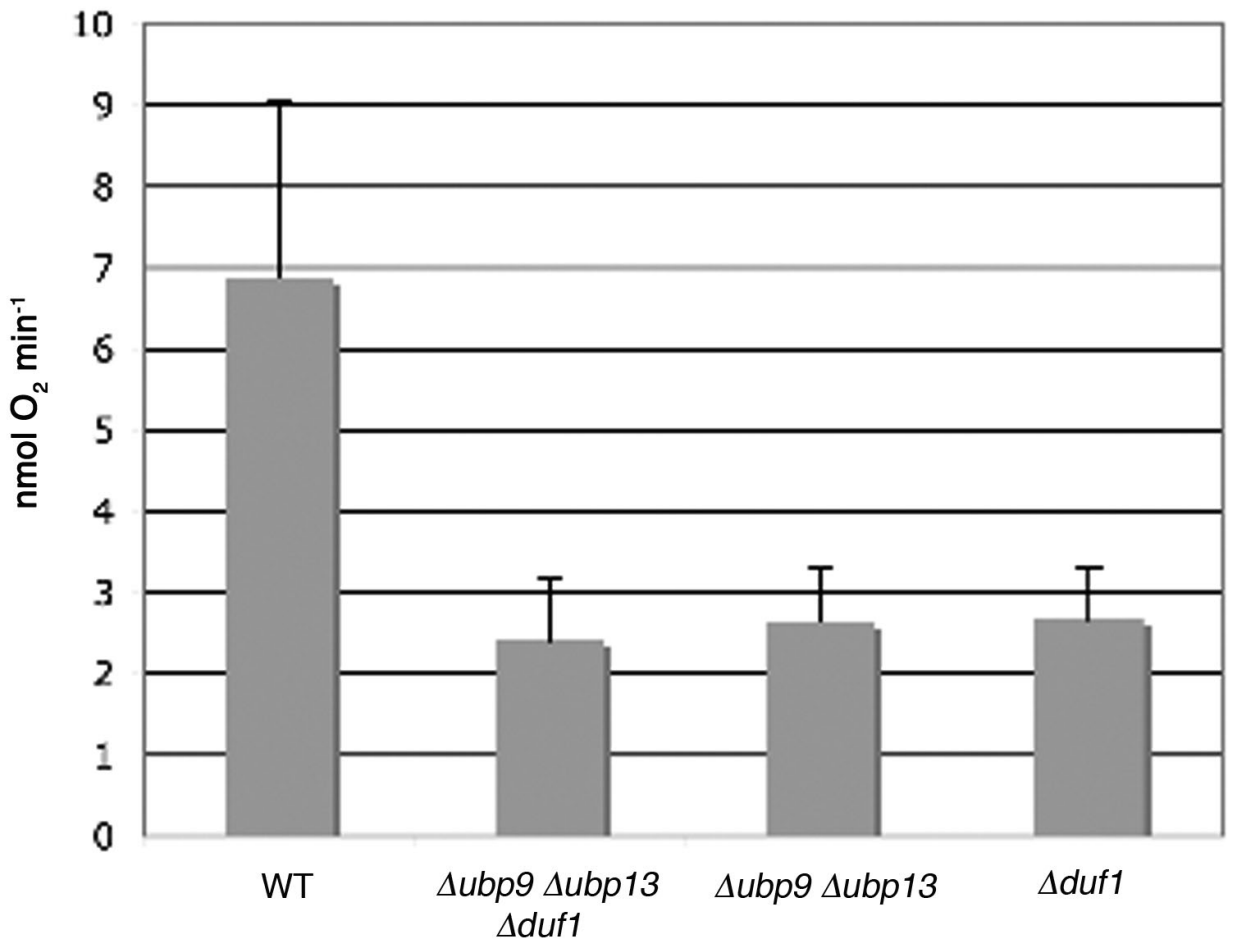
Δ*ubp9* Δ*ubp13*, Δ*duf1* and Δ*ubp9*Δ*ubp13*Δ*duf1* cells display defective respiration. Cells grown on galactose at 37°C to the exponential phase, were diluted in potassium buffer pH 7.2 and placed for a few hours at 37°C. Respiratory growth was then measured on entire cells, after the addition of 0.2% galactose, over a period of 7 min, with a Clark-Type electrode, as previously described [51].

Ubp9 and Ubp13 have both been reported to interact with Yol087c (Duf1, DUB-associated Factor 1) in large-scale proteome studies [31], [32]. Duf1 is a 125 kDa protein, a homolog of Bun107 from *S. pombe* and a distant homolog of the human Uaf1, both partners and activators of some Ubps [33], [34], [35]. Interestingly, the deletion of *DUF1* led to a respiratory phenotype similar to that observed in the Δ*ubp9* Δ*ubp13* double mutant (Fig. 2). The Δ*ubp9* Δ*ubp13* Δ*duf1* triple mutant did not display an aggravated phenotype, as shown by its respiratory growth (Fig. 2), and by the quantitative measurement of respiration at 37°C (Fig. 3), suggesting that the two Ubps and their putative partner are involved in the same mitochondrial function.

The respiratory growth defect of Δ*ubp9* Δ*ubp13* and Δ*duf1* strains was associated with a high incidence of *petite* colonies (Table 3). This was also the case for the deletion of *UBP13* alone, but not for the deletion of *UBP9,* suggesting that Ubp13 may play a more important role in mitochondrial function than Ubp9. As expected, the *petite* colonies obtained from Δ*ubp9* Δ*ubp13* and Δ*duf1* were not competent for respiration.

**Table 3:** Incidence of *petite* colonies in Δ*duf1* and Δ*ubp9* Δ*ubp13* cells.

Main genetic background	% *petite* colonies	Number of colonies counted
wild type	4%	158
Δ*duf1*	45%	221
Δ*ubp9* Δ*ubp13*	38%	210
Δ*ubp9*	4%	160
Δ*ubp13*	29%	102

For each strain, one respiratory competent colony was streaked on glucose medium. After 3 days of incubation at 30°C, the resulting colonies, of both small and regular size, were individually checked for their respiratory competence. Most of the small colonies were not respiration-competent and were counted as *petites* colonies. These data were duplicated and similar results were obtained.

In order to eliminate the possibility that the mitochondrial defect of Δ*ubp9* Δ*ubp13* and Δ*duf1* strains simply resulted from a general decrease in ubiquitin level, we checked the levels of free ubiquitin in stationary phase for cells grown on solid glucose medium, conditions in which Δ*ubp4* cells display marked ubiquitin depletion [24] (Supplemental Fig. S1). As expected, Δ*ubp4* cells contained low levels of free ubiquitin. By contrast, ubiquitin levels displayed no substantial difference in the Δ*ubp9* Δ*ubp13*, Δ*duf1,* and Δ*ubp9* Δ*ubp13* Δ*duf1* strains. The respiratory phenotype of these mutant cells is therefore not due to a general decrease in ubiquitin availability.

### Duf1 Physically Interacts with Ubp9 and Ubp13

Given the role of Ubp9, Ubp13 and Duf1 for normal mitochondria function, we first checked the localization of these three proteins. We found that these three proteins had similar distributions: GFP-tagged proteins were found mostly in the cytoplasm, in various growth conditions (data not shown), as described in databases for Ubp9 and Duf1 [36]. Biochemical fractionation of chromosomal-encoded HA-tagged proteins indicated that, in addition to the cytoplasmic soluble fraction, these proteins also display a membrane-bound fraction, possibly associated with mitochondria (Supplemental Fig. S2 B–C).

**Figure 4:**
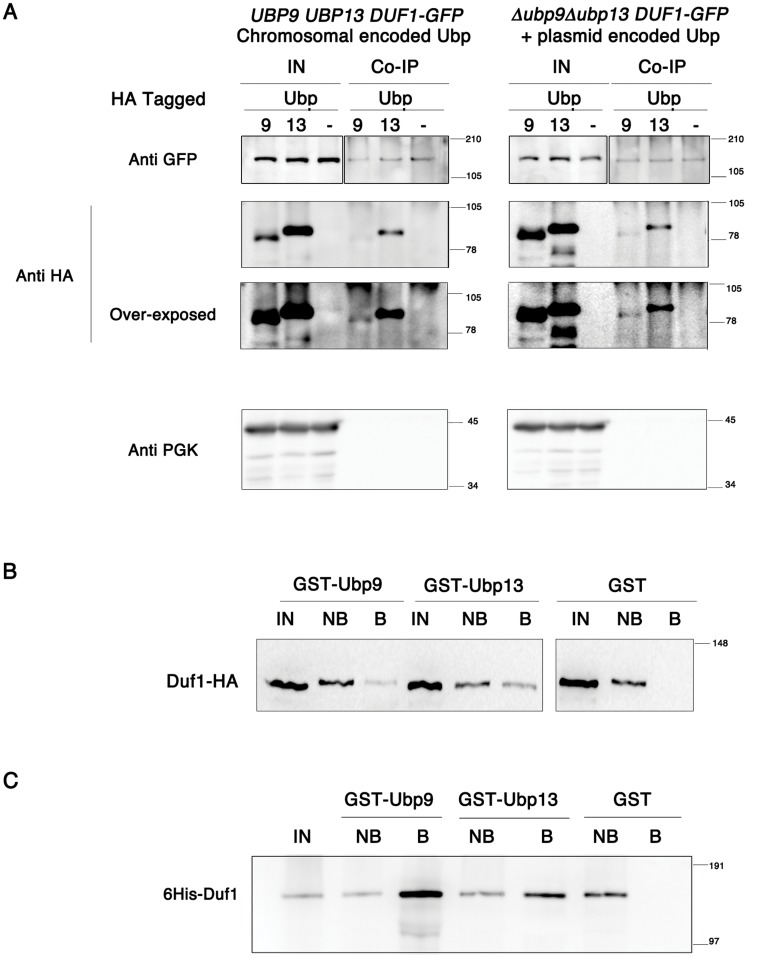
Both Ubp9 and Ubp13 interact with Duf1, a WD40 protein of unknown function. A. Ubp9 and Ubp13 coimmunoprecipitate with Duf1. Cells grown on lactate medium and expressing chromosome-encoded Duf1-GFP, and Ubp9-HA or Ubp13p-HA, were subjected to immunoprecipitation in native conditions using GFP antibodies. Total extracts (input, IN), and immunoprecipitates (IP) were tested by immunoblotting with anti-HA and anti-phopsphoglycerol kinase (PGK) antibodies. The same experiment was performed on Δ*ubp9* Δ*ubp13* expressing chromosomal-encoded Duf1-GFP (left) and plasmid-encoded Ubps (right). B. Ubp9 and Ubp13 interact with Duf1 in glutathione S-transferase (GST) pull-down assays. GST, GST-tagged Ubp9 and GST-tagged Ubp13 were purified with glutathione-Sepharose beads and incubated with extracts from cells producing Duf1-HA. Total extracts (IN), unbound (NB) and bound (B) fractions were analyzed by sodium dodecyl sulfate-polyacrylamide gel electrophoresis (SDS-PAGE) and immunoblotting with an anti-HA antibody. C. Ubp9 and Ubp13 interact directly with Duf1. The GST-pull down assay was performed as described in panel B, except that a bacterial extract producing 6His-Duf1 was used. 6His-Duf1 was detected with an anti-His antibody.

Large-scale proteome studies [31], [32] have indicated that both Ubp9 and Ubp13 interact with Duf1. We investigated this potential interaction both *in vivo* and *in vitro* (Fig. 4). Strains producing Duf1-GFP, and Ubp9-HA or Ubp13-HA tagged at the chromosomal locus were submitted to immunoprecipation in native conditions using anti-GFP antibody. Immunoprecipitates of Duf1-GFP retained both Ubp9-HA and Ubp13-HA (Fig. 4A left). The same data were observed in Δ*ubp9* Δ*ubp13* cells expressing plasmid-encoded Ubp9 or Ubp13 (Fig. 4A right). Thus, each Ubp interacts independently of the presence of the other with Duf1 *in* *vivo*.

For independent confirmation of the interaction, we carried out GST-pull down experiments with purified recombinant GST-tagged versions of Ubp9 or Ubp13 and yeast lysate prepared from cells expressing chromosome-encoded *DUF1-HA*. Both GST-Ubp9 and GST-Ubp13 were found to interact with Duf1, whereas GST alone did not (Fig. 4B). We investigated whether the interaction was direct or indirect, by carrying out GST-pull down assays with 6His-Duf1 produced in bacteria. Purified GST-tagged versions of Ubp9 and Ubp13 allowed the retention of bacterially produced 6His-Duf1, whereas GST alone did not (Fig. 4C). Overall, these data indicate that both Ubp9 and Ubp13 interact directly with Duf1, and that Ubp9/Duf1 and Ubp13/Duf1 form complexes *in vivo* and *in vitro*. In agreement with the presence of Duf1 in complex with Ubp9 or Ubp13, we observed that Duf1 is destabilized in the absence of its DUB partners, whether active or not (Material S1 and Supplemental Fig. S3).

### Duf1 Regulates the Enzymatic Activity of Ubp9 and Ubp13

**Figure 5:**
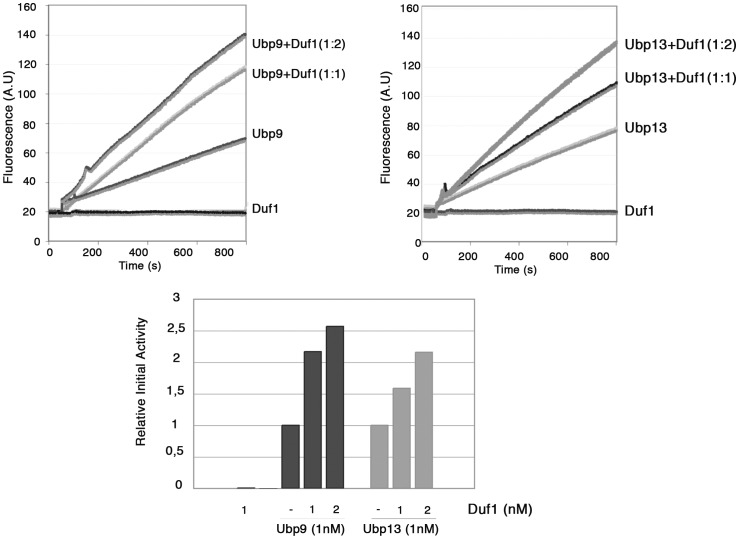
Duf1 is an activator of Ubp9 and Ubp13. A. The *in vitro* deubiquitylating activity of purified proteins was measured as described in Materials and Methods, with Ub-AMC as a substrate. The activity of purified wild-type Ubp9 and Ubp13 (1 nM), was measured as a function of time in the presence or absence of purified Duf1 (1 or 2 nM). For measurements in the presence of Duf1, the activity of Ubp9 and Ubp13 was determined after incubation for 10 min at room temperature in the presence of Duf1. B. The relative initial rates of activity of Ubp9 and Ubp13 in the presence and absence of various amounts of Duf1 are shown in arbitrary units. The data shown here correspond to one typical experiment. Independent experiments with other purification sets of Ubp9, Ubp13 and Duf1 yielded the same results.

It has been shown that some WD40 proteins can activate enzymatic activity of their DUB partners [33], [35]. We investigated whether Duf1 had a similar effect on the *in vitro* activity of Ubp9 and Ubp13. We purified GST-bound forms of Ubp9 and Ubp13, and removed the GST tag by protease cleavage. Both recombinant Ubp9 and Ubp13 displayed deubiquitylating enzyme activity with ubiquitin-AMC as the substrate (Fig. 5). Purified Duf1 displayed no deubiquitylating activity *per se*. A 10 min incubation of equal amounts of Ubp9 or Ubp13 with Duf1 resulted in an increase in the initial rate of activity. No such increase was observed when the control GST was added in similar or larger amounts (not shown). Doubling the amount of Duf1 further increased the deubiquitylating activity. Mixing the three proteins led to simple additivity of the DUB activities of activated Ubp9 and Ubp13 (data not shown). In conclusion, Ubp9 and Ubp13, which are active *in vitro* for the deubiquitylation of Ub-AMC, are both hyperactivated by the presence of their partner.

### Δ*ubp9* Δ*ubp13* and Δ*duf1* Cells Display Defective Biosynthesis of the Mitochondrial ATP Synthase Subunit Atp9 at the Level of *ATP9* Translation

**Figure 6:**
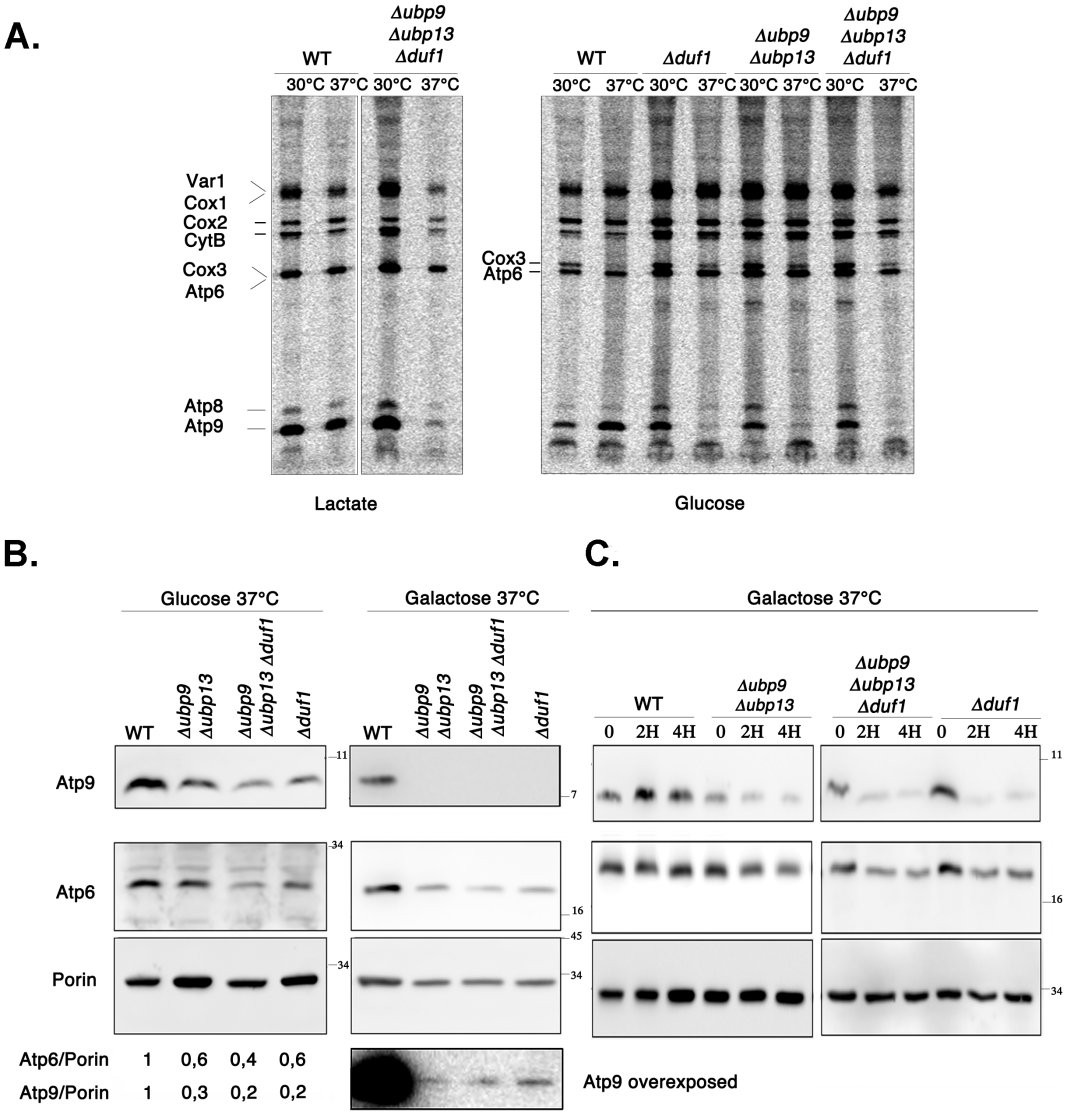
The deletion of *UBP9* and *UBP13* and the single deletion of *DUF1* impair the synthesis of the mitochondrial ATP synthase subunit Atp9 at 37°C. A. Pulse of mitochondrial translation. Wild-type or mutant cells were grown overnight at 30°C or 37°C in a complete medium with lactate or glucose as the carbon source. Cells were labeled by incubation for one hour at 30°C or 37°C in the presence of [S^35^] methionine and CHX (100 µg/ml). Proteins were precipitated and analyzed by electrophoresis in 12% polyacrylamide gels containing SDS, followed by autoradiography, as described in Materials and Methods. B. Wild-type and mutant cells were grown overnight at 37°C in complete medium with glucose or galactose as the carbon source. Aliquots of cells in the exponential growth phase were withdrawn, and analyzed by SDS-PAGE and immunoblotting with antibodies against Atp9, Atp6 and porin. The intensities of the bands were quantified by Image J, and the ratios Atp9/porin and Atp6/porin were calculated.C. Wild-type and mutant cells were grown overnight at 30°C in complete galactose medium. Cultures were diluted to an OD_600nm_ of 0.25 with the same medium and incubated at 37°C for the time (hours) indicated. Protein extracts were prepared and analyzed as in B.

Mitochondrial instability giving rise to *petite* colonies may have several causes: mutations in genes involved in mitochondrial DNA metabolism, or in genes controlling functions as diverse as iron homeostasis, fatty acid metabolism, mitochondrial morphology, mitochondrial translation, ATP synthase synthesis [23]. In our attempts to identify the potential origin of the “petite” phenotype of Δ*ubp9* Δ*ubp13 a*nd Δ*duf1* cells, we investigated the mitochondrial translation. Most of the >700 known yeast mitochondrial proteins are nuclear-encoded and imported into the mitochondria, with only eight proteins known to be encoded by the mitochondrial genome [22]: the ribosomal protein Var1, two polypeptides of the respiratory complex IV (Cox1, Cox2), Cox3, a subunit of cytochrome c oxidase, cytochrome *b* and three hydrophobic subunits of the F0 part of the ATP synthase (complex V) located in the inner mitochondrial membrane (Atp6, Atp8 and Atp9) [22]. We monitored mitochondrial translation by *in vivo* pulse labeling of mitochondrial translation products with [^35^S]methionine in the presence of cycloheximide, which specifically inhibits cytoplasmic but not mitochondrial translation. Labeled proteins synthesized over a period of one hour were analyzed by SDS-PAGE and autoradiography (Fig. 6A, left panel). The experiment was performed with the wild type, and with the various mutant cells, grown at 30°C or 37°C. Δ*ubp9* Δ*ubp13* Δ*duf1* triple deletant displayed a profile of mitochondrion-synthesized proteins identical to that of wild-type cells after growth in lactate medium at 30°C. Growth at 37°C in lactate medium led to a slight decrease in the synthesis of some mitochondrial-encoded proteins in both wild type cells and Δ*ubp9* Δ*ubp13* Δ*duf1* triple deletant. In addition, these mutant cells displayed a striking drop in the synthesis of Atp9, the polymeric membrane-bound subunit of the mitochondrial inner membrane that forms, together with Atp6, the proton channel of the mitochondrial ATP synthase.

We checked whether the defect observed for Atp9 was dependent on the growth medium, and whether it was also found in Δ*ubp9* Δ*ubp13* or Δ*duf1* cells. We thus studied mitochondrial translation for Δ*ubp9* Δ*ubp13*, Δ*duf1* mutants and for Δ*ubp9* Δ*ubp13* Δ*duf1* mutants grown at 30°C or 37°C on glucose, a fermentable medium in which respiration is not required. The mutant cells displayed normal mitochondrial protein synthesis after growth at 30°C, whereas, at 37°C, Atp9 was barely detectable in the three mutants (Fig. 6A, right panel), in the absence of a marked change in the levels of other mitochondrion-encoded proteins (Fig. 6A and Supplemental Fig. S4). Thus, our results demonstrate a specific change in the level of Atp9 translation in the absence of Duf1 or Ubp9/Ubp13.

We investigated whether the translation defect of Atp9 affected the steady-state level of Atp9, by carrying out western blotting to analyze the amounts of this protein in cells grown using as carbon source either glucose, exclusively fermentable, or galactose, used by fermentation and respiration simultaneously in exponentially growing cells [37]. In both cases, Atp9 levels were low in Δ*ubp9* Δ*ubp13*, Δ*duf1* and Δ*ubp9* Δ*ubp13* Δ*duf1* mutants after overnight growth at 37°C (Fig. 6B). This decrease in Atp9 level was particularly striking for mutant cells grown in galactose medium. We monitored the kinetics of Atp9 levels at 37°C, in wild-type, Δ*ubp9* Δ*ubp13,* Δ*duf1,* and Δ*ubp9* Δ*ubp13* Δ*duf1* cells (Fig. 6C) grown in galactose-containing medium at 30°C and then shifted to 37°C for various periods of time. Atp9 levels were already clearly lower than those in wild-type cells after 2 hours at 37°C (Fig. 6C *).* Mutant cells also had slightly lower levels of the mtDNA-encoded Atp6, an effect probably secondary to the decrease in Atp9 levels, as already described for some *atp9* mutants [38].

Thus, the deletion of *UBP9/UBP13* or *DUF1* specifically affects the biosynthesis of Atp9 at 37°C, and this defect results in lower steady-state levels of this subunit in mitochondria. ATP synthase F0 deficiency has been shown to result in impaired mitochondrial genome integrity [23], so the observed decrease in Atp9 levels likely provides an explanation for the increase in frequency of *petite* and respiratory deficiency phenotypes in Δ*ubp9* Δ*ubp13* and Δ*duf1* mutant cells.

Biogenesis of a functional ATP synthase, which consists of both nuclear-encoded and mitochondrial-encoded subunits, is an elaborate process, and lesion in ATP synthase can result from defective function of a number of nuclear genes required at pre- and post-translational stages of the assembly pathway [39]. Many accessory factors were described to be required for synthesis or assembly of F1 and F0 moieties of ATP synthase including for the synthesis of the F0 subunit Atp9. The defective biogenesis of Atp9 we observed could potentially result from defects in *ATP9* mRNA biogenesis, maturation, or translation. We first checked the level of *ATP9* mRNA in wild-type and mutant cells grown overnight in galactose- or glucose-containing medium at 30° or 37°C (Supplemental Fig. S5). We compared the relative amounts of *ATP9* mRNA and of control nuclear-encoded *ACT1* mRNA or mitochondrion-encoded *ATP6* mRNA. *ATP9* mRNA levels were not significantly lower in the mutants than in wild-type cells. The limited variation in *ATP9* mRNA levels observed cannot account for the striking differences in Atp9 protein level. We then investigated whether *ATP9* mRNA processing was affected in mutant cells at 37°C, by performing 5′ mRNA extension (Supplemental Fig. S6). Several extension products were detected, ranging from 150 to 300 upstream nucleotides, demonstrating the non-homogeneity of the 5′-termini of *ATP9* mRNA. However, no significant difference between wild-type and mutant cells was observed at either growth temperature or with the different carbon sources. Thus, 5′-UTR maturation of the *ATP9* mRNA was not responsible for the observed deficiency.The overall results indicate that the DUB complex controls *ATP9* expression at the level of mRNA translation.

## Discussion

The ubiquitin proteasome system has already been reported to be involved in multiple mitochondrial functions [5], but the role of DUBs in these processes is poorly documented. We used a systematic screen to identify the yeast UBPs required for normal mitochondrial function and then focused on the role of Ubp13, the closely related Ubp9, and their binding partner, the WD40 protein Duf1. The deletion of both *UBP9* and *UBP13,* or of *DUF1* alone, resulted in similar respiratory growth defects, associated with instability of the mitochondrial genome, indicating that Ubp9, Ubp13 and Duf1 act in the same mitochondrial pathway. We report here that Ubp9, Ubp13 and Duf1 regulate the expression of the mitochondrial ATP synthase subunit 9 at the level of translation.

DUBs have been described to have multiple partners that play a role in substrate recognition, localization to various cellular compartments, or activation (reviewed in [4]). According to databases, many yeast Ubps appear to interact with WD-repeat proteins [34]. A global proteomic analysis of human DUBs and their associated protein complexes revealed that 36% of DUBs are associated with WD40 proteins [40], showing that the association of UBPs with WD40-containing proteins is a very general process. However, the functions of the WD40 interacting proteins have been documented in only a few cases. The first description of a functional link between a DUB and a WD40 protein was the genetic evidence that the DUB CreB of *Aspergillus nidulans* interacts with the WD40 protein CreC, and that they are both involved in carbon catabolite repression [41], [42]. It was then shown that several human WD40 proteins interact with and activate DUB partners [34] [33], sometimes with two WD40 proteins required for optimal activity [43]. More recently, a global analysis of the localization and interaction network of DUBs in *S. pombe* has shown that Ubp9*^S.p^* (ortholog of Sc Ubp9) interacts with two WD40 proteins (including Bun107, an ortholog of Duf1), both of which are required for *in vitro* Ub-AMC deubiquitylation by a Tap-tagged Ubp9 purified after *in vivo* expression [35]. We report here that Duf1 interacts with and activates two DUBs, Ubp9 and Ubp13 in *S. cerevisiae*. This situation appears to differ from that of Usp1/UAF1 or Ubp9^sp^ and WD40 partners, because the recombinant Ubp9 and Ubp13 are already active in the absence of Duf1, at least *in vitro,* with the substrate Ub-AMC, and are over-activated in the presence of Duf1. However, the respiratory phenotype of the Δ*ubp9* Δ*ubp13* Δ*duf1* triple mutant is not more severe than that of Δ*duf1* cells, so it is possible that Ubp9 and Ubp13 can deubiquitylate their physiological substrate, which is important for their mitochondrial function, only in the presence of their Duf1 partner.

Many DUBs bind ubiquitin with a reasonable affinity, but others have little affinity for ubiquitin. They therefore interact with their ubiquitylated substrate through associations with partners [4]. It was recently shown that some WD40 domains, including that of Duf1, interact with ubiquitin [44]. In particular, it has been shown that the amino acids of ubiquitin involved in Duf1 binding largely overlap with those involved in binding to the WD40-containing F–box protein Cdc4 [44]. We observed that Duf1 ubiquitin binding was not restricted to the isolated WD40 domains, but was instead a property of the entire Duf1 protein (data not shown). The precise role of ubiquitin binding within the Ubp/Duf1 complex remains to be deciphered. It may play a role in the recognition of physiological substrates. The binding of Cdc4 to ubiquitin has been shown to be required for its ubiquitin-dependent turnover [44], but the underlying mechanism remains unknown. Whether ubiquitin binding plays a role in the ubiquitin-dependent turnover of Duf1 is also an open question.

The precise physiological functions of DUBs remain poorly documented. In budding yeast, none of the DUBs belonging to the UBP subfamily is essential for viability [2]. In *S. pombe*, the deletion of five DUBs, including Ubp9^sp^, was required to observe a growth phenotype [35]. Very little is known about DUBs and mitochondria. The only human DUB known to be required for mitochondrial function is USP30 [21], and no yeast DUBs have yet been shown to be essential for normal mitochondrial function. We report here that the redundant Ubp9, Ubp13 and their partner, Duf1, are required for normal respiration, a phenotype already detected at 30°C and exacerbated at 37°C. Mitochondrial oxidative phosphorylation is catalyzed by the respiratory chain and the proton-translocating ATP synthase. This multicomponent enzyme consists of a hydrophilic F1 moiety containing the nucleotide-binding and catalytic site, and a hydrophobic F0 moiety containing the proton channel, made of 10 copies of subunit 9 arranged in a ring, and one copy of Atp6 [22] [39]. The observation that Ubp9, Ubp13 and Duf1 are required for the biogenesis of Atp9 at 37°C provides clues to the origin of the respiratory deficiency of Δ*ubp9* Δ*ubp13* and Δ*duf1* cells at 37°C. A high degree of mitochondrial genome instability, resulting in the formation of *petite* colonies, was indeed observed in cells lacking the genes encoding F0 subunits or proteins required for synthesis or assembly of the F0 complex [23].

Our discovery that Ubp9, Ubp13 and Duf1 are required for the biogenesis of Atp9 provides insight into the formation of this essential F0 subunit. Atp9 is one of the few mitochondrial proteins encoded by the mitochondrial genome in yeast (it is of nuclear origin in mammals). As for other mitochondrion-encoded ATP synthase subunits [39], Atp9 biogenesis is a highly regulated process, and several factors involved in this process have already been described. (reviewed in [39]). We observed no significant alteration in the amount of *ATP9* mRNA or its processing in Δ*ubp9* Δ*ubp13* and Δ*duf1* mutant cells. By contrast, at 37°C, the mutants had low levels of newly synthesized Atp9, indicating that the final step in the regulation process appears to be the control of the translation of *ATP9* mRNA.

Although future experiments are now required to identify the potential common target(s) of the Duf1/Ubp9/Ubp13 deubiquitylating complexes on ATP9 translation, this work provides the first evidence of a role for the ubiquitin system, and a DUB complex in particular, in the regulation of mitochondrial functions through a tight control of ATP synthase synthesis.

## Materials and Methods

### Construction, Manipulation and Growth of Yeast Strains

All the yeast strains used in this study are listed in Table 1. All strains are derivatives of BY4741/2, except for YDB123 and YDB124 (parental cells WCG4a, [45]). Null alleles of yeast genes were constructed by PCR-based homologous recombination using pFA6a-natNT2 [46] for the deletion of *PDR5*, and pF6a-kanMX6 [47] for the deletion of the other genes. When required, the yEGFP tag was amplified from pYM44 [46] and a triple HA-tag was amplified from pFA6a-3HA-His3MX6 [47]. The YDBn and DBn strains were obtained by integrative transformation and/or meiosis following appropriate crosses. Addition of the HA tag to Duf1 and Ubp13 generated no particular phenotype on respiratory medium and the incidence of *petite* colonies was similar to that for the wild type. Cells were transformed by the lithium acetate procedure [48]. All experiments were performed on cells collected in exponential growth phase, unless otherwise indicated. Glucose (2%) or galactose (2%) was used as the fermentable substrate and ethanol/glycerol (2%) or lactate (2%) was used as the respiratory substrate.

### Plasmids

The plasmids for the expression of *GST-UBP9* and *GST-UBP13* in bacteria were generated by inserting a *Bam*HI-*Xho*I PCR fragment prepared from the genomic DNA of BY4741 cells into pGEX4T-1 or pGEX6P-1 (Amersham Biosciences). The plasmid for the bacterial expression of 6His-Duf1 was obtained by inserting the *Sal*I-*Not*I *DUF1* PCR fragment prepared from the genomic DNA of BY4741 cells into pET28B (Novagen). The centromeric plasmids pFL38-*UBP9-3HA* and pFL38-*UBP13-3HA* were constructed by inserting PCR-amplified tagged chromosomal Ubp9-3HA or Ubp13-3HA genes, under the control of their endogenous promoter, into pFL38 (ARS/CEN, *URA3*). We generated pFL38/pUL9-*UBP13-3HA* (ARS/CEN, *LEU2)* by gap repair in yeast. For this purpose, cells were cotransformed with *Nco*I-linearized pFL38*-UBP13-3HA* and *Sma*I-digested pUL9, as previously described [49], using pUL9, an *URA3-LEU2* plasmid converter containing the *LEU2* marker surrounded by two regions of homology with *URA3* [49]. For mutagenesis, the QuickChange Lightning Site-Directed Mutagenesis Kit (Stratagene, Lajolla, USA) was used, according to the manufacturer’s instructions, to generate point mutations in the regions corresponding to the catalytic sites of *UBP9* (Cys143Ser) and *UBP13* (Cys149Ser) [50] in pFL38-*UBP9-3HA* or pFL38/pUL9-*UBP13-3HA* (ARS/CEN, *LEU2).* The resulting plasmids were named pFL38-*UBP9C/S-3HA* and pFL36-*UBP13C/S-3HA*. A *GST-UBP9C/S* construct was also generated from a *pGEX6P-1/GST-UBP* plasmid.

### Oxygen Consumption

Yeast strains were grown in YPD medium. The respiratory activity of whole cells, prepared as 50% (w/v) suspensions in 0.1 m potassium phosphate buffer, pH 7.2, was evaluated by an oxypolarographic method, as previously described [51].

### Protein Extracts, Immunoprecipitation and Coimmunoprecipitation Experiments

For western blots, total protein extracts were prepared from three OD_600nm_ units of yeast, by the NaOH-TCA lysis technique, as previously described [52]. Aliquots corresponding to 0.2 OD_600nm_ units (unless otherwise indicated) were analyzed by western blotting after SDS-PAGE in a 10% polyacrylamide gel. For immunoprecipitation, cells grown on galactose or glucose (40 OD_600_ units) were harvested by centrifugation at 4°C, and resuspended in 1.5 ml of cold lysis buffer (TNE buffer: 100 mM Tris–HCl, pH 7.5; 150 mM NaCl; 5 mM EDTA plus a mixture of protease inhibitors – Complete from Roche Diagnostics) and 25 mM freshly prepared N-ethylmaleimide, for immunoprecipitation in denaturing conditions, or in lysis buffer (50 mM Hepes-KOH, pH 7.5, 150 mM NaCl, 0.1% NP40, 10% glycerol, 1 mM EDTA, MG132, 1 mM PMSF and protease-inhibitor cocktail), for coimmunoprecipitation. Cells were then disrupted by vortexing with beads or with a ‘One Shot’ Cell Disrupter and centrifuged twice (3000 *g* for 3 minutes at 4°C) to remove unbroken cells. For coimmunoprecipitation, the lysate was incubated for 1 h at 4°C with GammaBind-Sepharose beads (GE, Healthcare) that had previously been incubated with a monoclonal anti-HA antibody (Santa Cruz). The beads were washed three times with lysis buffer and proteins were eluted in SDS sample buffer for 10 minutes at 95°C. For immunoprecipitation the resulting lysate was subjected to precipitation by adding TCA (10%) and incubating on ice for 10 minutes. After centrifugation the pellet was resuspended in 60 µl of SDS sample buffer without 2-mercaptoethanol and incubated for 10 minutes at 95°C. We added 0.6 ml of TNET buffer (TNE +1% Triton X-100) and the mixture was centrifuged at 4°C for 30 minutes at 12,000×*g*. Antibodies were added to the supernatant, which was incubated for 30 minutes at 4°C, with shaking. We then added 50 µl of freshly prepared Protein G Sepharose beads (Gamma Bind G Sepharose, Amersham Pharmacia) and incubated the mixture overnight at 4°C. The pellets were washed four times with 1 ml of TNET buffer, resuspended in sample buffer and heated for 10 minutes at 95°C for SDS–PAGE and immunoblotting analysis.

### Production of GST-tagged Proteins

*Escherichia coli* BL21 strains expressing GST, GST-*UBP9*, GST-*UBP13*, or 6HIS-*DUF1* were cultured at 37°C until an OD_600_ of 0.6 was reached. They were then subjected to cold and chemical shocks (treatment for 10 min at 4°C in the presence of ethanol 2%) and gene expression was induced by overnight incubation with 0.3 mM IPTG at 18°C for GST-Ubps or at 23°C for 6His-Duf1. Fusion proteins were isolated according to the kit manufacturer’s instructions. GST-Ubps were purified on glutathione-Sepharose 4B beads (Amersham Biosciences), and 6His-Duf1 was purified on Ni^2+^-NTA Superflow resin (Qiagen Inc.). For the *in vitro* deubiquitylation assay, GST-Ubp9, GST-Ubp13, and 6HIS-Duf1 were produced and purified on glutathione or nickel beads. The 6His-Duf1 was eluted from the nickel beads, whereas the GST-tag was cleaved from the beads by overnight incubation with thrombin (Amersham) or incubation for four hours at 4°C in the presence of the Prescission protease (Amersham). Ubp9 and Ubp13 were further purified by gel filtration (Superdex 200) with 50 mM Tris-HCl pH 7.5, 200 mM NaCl, 10% glycerol, 2 mM DTT.

### GST-pull Down

Yeast cells producing Duf1-HA and growing exponentially on glucose or galactose complete medium were harvested and the pellet was resuspended in 1.5 ml lysis buffer (50 mM Tris pH 7.4, 300 mM NaCl, 10 mM MgCl_2_, 0.1% Triton X-100, 1 mM DTT, 10% glycerol plus protease inhibitors). Cells were disrupted in a ‘One Shot’ Cell Disrupter and the extract was centrifuged twice (3,000×*g*, for 3 minutes each, at 4°C) to remove unbroken cells, yielding lysate (Input, IN). The pull-down reaction was performed in a final volume of 750 µl, with 160 µg GST or 40 µg GST-Ubp9/GST-Ubp13, and either a lysate of yeast cells producing Duf1-HA or a bacterial lysate of cells expressing 6His-Duf1. The reaction mixture was incubated for 1 h at 4°C with gentle shaking and then centrifuged. An aliquot (50 µl) of the supernatant corresponding to the unbound fraction (NB) was collected. The beads were washed eight times with 750 µl of lysis buffer and proteins bound (B) to the beads were eluted with 75 µl SDS sample buffer and heated for 10 minutes at 95°C. The lysate (IN) and the unbound (NB) fraction were heated in 50 µl of SDS sample buffer.

### *In Vitro* Enzymatic Deubiquitylation Assay

The assay was performed by adding Ubiquitin-AMC (ubiquitin-7-amido-4-methylcoumarin; Boston Biochem) 60 seconds after beginning to monitor activity, in a total volume of 600 µl. The assay buffer was 50 mM Tris/HCl pH 8.0, 100 mM NaCl, 1 mM EDTA, 5 mM DTT, 0.01% Tween-20. Protein concentration was determined with a Nanodrop 1000 machine (Thermoscience). Fluorescence was monitored in a QuantaMaster30 (Photon Technology International) for at least 800 seconds at room temperature.

### Mitochondrial Translation and Western-blot Analysis

Pulse chase mitochondrial translation was performed as previously described [53] in the presence of ^35^S-methionine (NEN, 400 Ci/mmole) and cycloheximide. The proteins generated were extracted in NaOH, precipitated in TCA and washed in water before separation by denaturing SDS-PAGE in a 12 or 20% polyacrylamide gel and analysis by autoradiography with a Typhoon-Trio phosphor imager and ImageQuant software (GE Healthcare). In parallel, for quantification, an aliquot of extracted proteins was subjected to SDS-PAGE in a 10% polyacrylamide gel, with quantification by Ponceau staining and western immunoanalysis with antibodies against Atp9, Atp6 or porin.

### Antibodies

Polyclonal antibodies against cytochrome *b*_2_ (B. Guiard, Gif-sur-Yvette, France), AAC (gift from N. Pfanner, Freiburg, Germany), Atp6 and Atp9 (gift from J-P. Di Rago and D Brèthes), and Sss1 (gift from F. Kepes, Saclay, France) and monoclonal antibodies against HA and 6His epitopes (Santa Cruz Biotechnology), GFP (Roche Diagnostics), phosphoglycerol kinase (PGK), porin, Vph1 (Molecular Probes), and ubiquitin (Ub-HRP conjugate, Santa-Cruz Biotechnology) were used for the immunodetection of immobilized proteins. Horseradish peroxidase-conjugated anti-mouse immunoglobulin G was used as the secondary antibody (Sigma) and was detected by enhanced chemiluminescence (ECL).

## Supporting Information

Figure S1 **The respiratory phenotype of** Δ***ubp9*** Δ***ubp13*** **and** Δ***duf1*** **mutants is not due to a general decrease in free ubiquitin levels.** Crude extracts were prepared from cells grown on solid glucose, under the conditions described in Fig. 1 (stationary phase). Extracts from equivalent numbers of cells (based on OD units) were separated in a 5% to 16% MES polyacrylamide gradient gel (Invitrogen) and the bands transferred to PVDF membrane. A monoclonal anti-ubiquitin antibody from Zymed was used to detect free ubiquitin (Ub), and the immunodetection of PGK was used as a loading control. Ub* corresponds to a shorter exposure. The monoubiquitin signal was quantified with ImageJ software, and normalized with respect to the PGK signal. The abundance of monoubiquitin in the various strains relative to that in wild-type cells is indicated below the lanes.
(TIF)

Figure S2 **Ubp9, Ubp13 and Duf1 display dual localization in soluble and membrane-bound fractions.** A. Protoplasts were prepared from cells grown on galactose medium and expressing chromosome-encoded Ubp9-HA (YDB105), Ubp13-HA (YDB106), Duf1-HA (YDB107) or Ubp16-GFP. Aliquots of 13,000 *g* pellets (P13) and supernatants (S13) corresponding to equivalent numbers of cells were analyzed by SDS-PAGE and immunoblotting with antibodies against HA or GFP, PGK and porin. B. Ubp9, Ubp13 and Duf1 are found in fractions enriched in mitochondria. Fractions enriched in mitochondria were prepared from cells grown on lactate medium at 30°C and expressing chromosome-encoded Ubp9-GFP, Ubp13-HA (DB122-1D), or Duf1-HA (YDB107). Equal amounts of protein (80 µg) from the post-mitochondrial supernatant (PMS), crude mitochondria (mit1) and mitochondria further purified on a sucrose gradient (mit2) were loaded onto gels and analyzed by SDS-PAGE. Immunodetection was carried out with antibodies against porin, PGK, Sss1 and Vph1, as markers of the mitochondrial, cytosolic, ER and vacuolar compartments, respectively. Duf1-HA displayed some degradation products in mit1 fractions. C. Ubp9, Ubp13, and Duf1 are membrane-bound proteins. Fractions enriched in mitochondria (mit2) from cells producing HA-tagged Ubp9, Ubp13 or Duf1 (YDB105, YDB106 and YDB107) were sonicated on ice. Samples were left untreated (T) or subjected to ultracentrifugation at 100,000 *g* (S100, supernatant; P100, pellet) and then analyzed by SDS-PAGE and immunoblotting. Immunodetection was carried out with antibodies against HA, AAC and cytochrome *b*_2_ (cyt *b*_2_) as markers of the mitochondrial membrane and soluble fraction, respectively.
(TIF)

Figure S3 **Duf1 is an unstable, ubiquitylated protein, further destabilized in the absence of its two protein partners, Ubp9 and Ubp13.** A. Steady-state levels of Duf1 decrease in the Δ*ubp9* Δ*ubp13* double mutant. Crude extracts prepared from cells expressing chromosome-encoded Duf1-HA in wild-type, Δ*ubp9*, Δ*ubp13* and Δ*ubp9* Δ*ubp13* backgrounds were grown on glucose-rich medium at 30°C and analyzed by western blotting with HA and PGK antibodies. B. The half-life of Duf1 is modified in the Δ*ubp9* Δ*ubp13* double mutant. Cells expressing Duf1-HA in wild-type or Δ*ubp9* Δ*ubp13* backgrounds were grown in glucose-rich medium at 30°C, and crude extracts were prepared at various times after the addition of cycloheximide (100 µg/ml). The stability of Duf1-HA was then monitored by SDS-PAGE and immunoblotting, with PGK antibody as a loading control. C. Duf1 is stabilized in the *pre1-1 pre2-2* mutant cells. Crude extracts were prepared from cells expressing chromosome-encoded Duf1-HA gene, in wild-type or *pre1-1 pre2-2* backgrounds, and growing exponentially in glucose-rich medium at 30°C, or after incubation for 1 h at 37°C. The steady-state level of Duf1-HA was then monitored by SDS-PAGE and immunoblotting, with anti-PGK antibody as a loading control. D. Duf1 is ubiquitylated. Cells growing exponentially on galactose-rich medium and producing chromosome-encoded Duf1-HA were subjected to immunoprecipitation in denaturing conditions with an anti-HA antibody. Input fractions (Pre), unbound material (Post) and immunoprecipitates (IP) were immunoblotted with the anti-HA and anti-ubiquitin antibodies. E. Duf1 stability depends on the physical presence of Ubp9 and Ubp13. Δ*ubp9*Δ*ubp13* cells producing chromosome-encoded Duf1-HA either non transformed (-), or transformed with control empty plasmids (Ø), pFL38-UBP9C/S-HA plus pFL36-UBP13C/S-HA (C/S), or pFL38-UBP9-HA plus pFL38/pUL9-UBP13-HA (WT) were grown in glucose rich medium. Protein extracts prepared from wild-type cells expressing chromosome-encoded Duf1-HA were also analyzed (left first lane). Protein extracts were analyzed by western blotting with anti-HA antibodies, with PGK antibody as a loading control.
(TIF)

Figure S4 **The deletion of** ***UBP9*** **and** ***UBP13*** **and the single deletion of** ***DUF1*** **impair the synthesis of the mitochondrial ATP synthase subunit Atp9 at 37°C.** The amount of each mitochondrial genome-encoded protein in mutant cells was determined relative to that in wild-type cells in the experiment described in Fig. 6.
(TIF)

Figure S5 **Northern analysis of yeast mRNAs.** (A) Autoradiographs of washed filters for RNA extracted from yeast and separated in denaturing agarose gels are presented. Yeast strains were cultured with either glucose or galactose as the carbon source (as indicated above the autoradiographs) at two temperatures, 30°C and 37°C (as indicated above the autoradiographs). The samples are as follows: (1) Δ*duf1,* (2) WT (wild type), (3) Δ*duf1,* Δ*ubp13,* Δ*ubp9,* (4) Δ*ubp13,* Δ*ubp9* (as indicated below the autoradiographs). The probes used for hybridization are indicated at the left of the autoradiographs: ACT, actin; *ATP6* and *ATP9*. (B) Relative quantification results: the ratios of different hybridization signals are presented in the table. The 30°C/37°C ratios for the WT strain were taken for 1 in each series.
(TIF)

Figure S6 **Analysis of** ***ATP9*** **mRNA 5′-end maturation by primer extension.** Autoradiographs of 10% polyacrylamide denaturing SDS-PAGE gels on which the products of primer extension were separated. Yeast strains were cultured in the presence of either glucose or lactate as the carbon source (as indicated above the lanes) at two temperatures, 30°C and 37°C. Two different amounts of yeast RNA were tested: 20 or 40 µg per assay (as indicated above the autoradiographs). The samples are as follows: (1) WT (wild type), 30°C; (2) WT, 37°C; (3) Δ*duf1,* 30°C; (4) Δ*duf1,* 37°C; (5) Δ*ubp13* Δ*ubp9,* 30°C; (6) Δ*ubp13* Δ*ubp9,* 37°C; (7) Δ*duf1* Δ*ubp13* Δ*ubp9,* 30°C; (8) Δ*duf1* Δ*ubp13* Δ*ubp9,* 37°C. “L” - labeled ladder from the primer extension kit (Promega). The size of a selection of fragments is indicated to the left of the panels. “C” the control extension assay obtained with RNA and the primer supplied in the kit (expected size: 84 nucleotides). On the right, the extension products obtained with yeast RNA or control RNAs are indicated by the arrows.
(TIF)

Material S1 **Supplementary Materials and Methods.**
(DOC)
