# Supplementary figures and images for: A Deubiquitylating Complex Required for Neosynthesis of a Yeast Mitochondrial ATP Synthase Subunit
Source: PLoS One. 2012 Jun 19;7(6):e38071. doi: 10.1371/journal.pone.0038071 (PMC3378586; doi:10.1371/journal.pone.0038071)

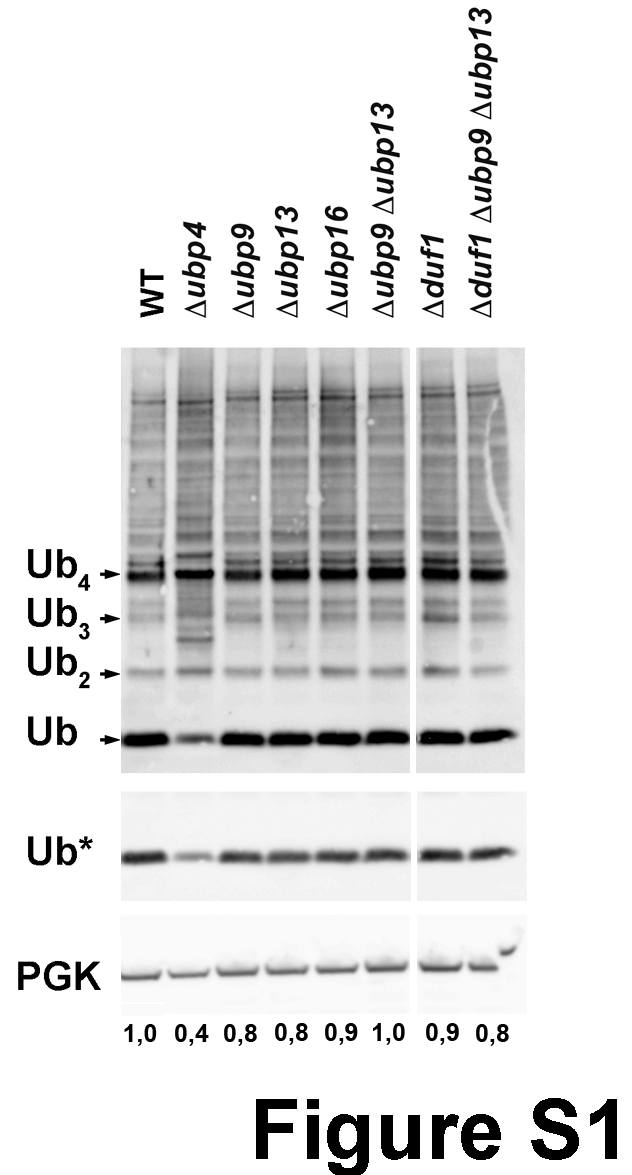

Supplement: Figure S1 — The respiratory phenotype of Δubp9 Δubp13 and Δduf1 mutants is not due to a general decrease in free ubiquitin levels. Crude extracts were prepared from cells grown on solid glucose, under the conditions described in Fig. 1 (stationary phase). Extracts from equivalent numbers of cells (based on OD units) were separated in a 5% to 16% MES polyacrylamide gradient gel (Invitrogen) and the bands transferred to PVDF membrane. A monoclonal anti-ubiquitin antibody from Zymed was used to detect free ubiquitin (Ub), and the immunodetection of PGK was used as a loading control. Ub* corresponds to a shorter exposure. The monoubiquitin signal was quantified with ImageJ software, and normalized with respect to the PGK signal. The abundance of monoubiquitin in the various strains relative to that in wild-type cells is indicated below the lanes. (TIF) [file pone.0038071.s001.tif]

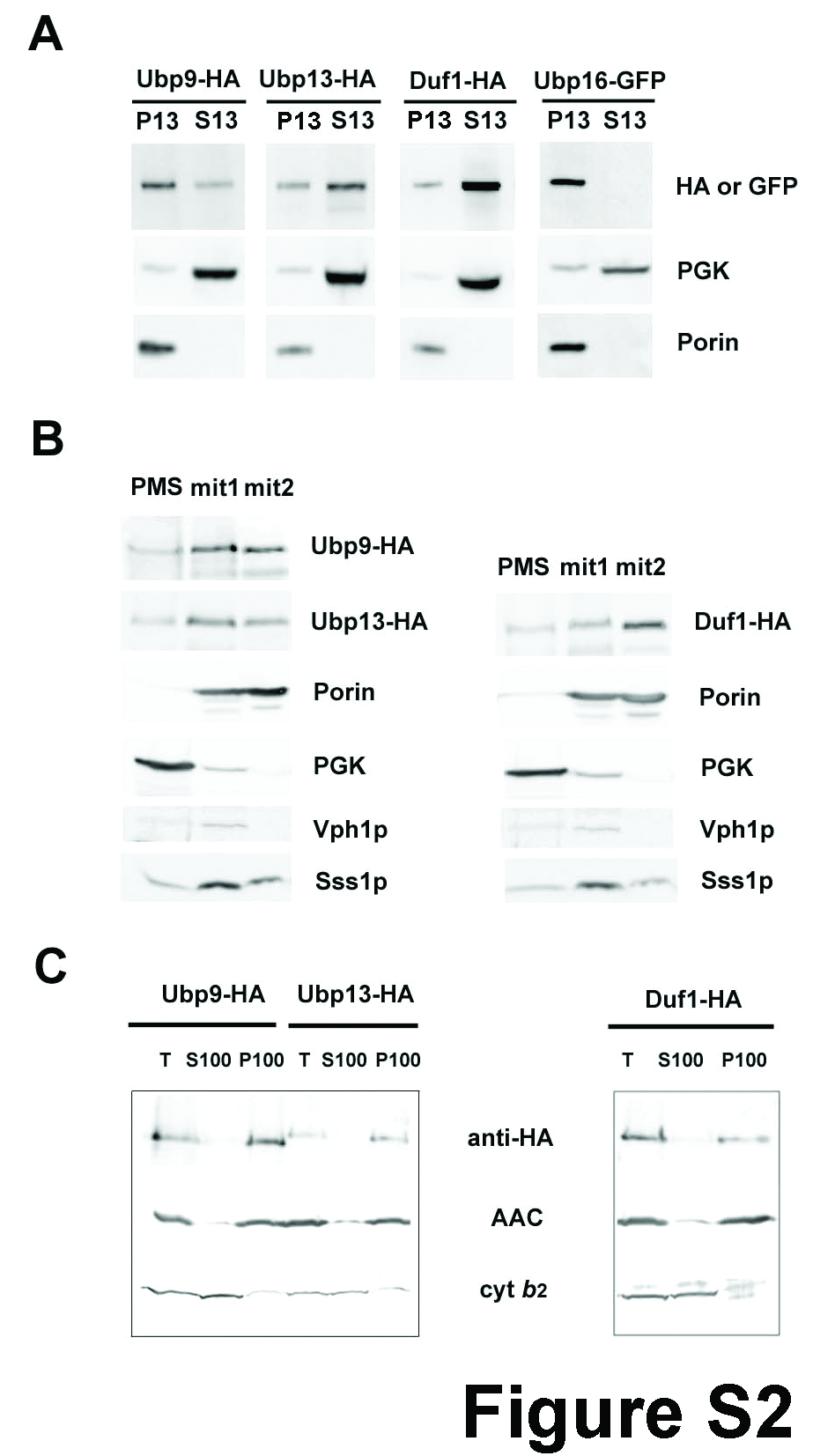

Supplement: Figure S2 — Ubp9, Ubp13 and Duf1 display dual localization in soluble and membrane-bound fractions. A. Protoplasts were prepared from cells grown on galactose medium and expressing chromosome-encoded Ubp9-HA (YDB105), Ubp13-HA (YDB106), Duf1-HA (YDB107) or Ubp16-GFP. Aliquots of 13,000 g pellets (P13) and supernatants (S13) corresponding to equivalent numbers of cells were analyzed by SDS-PAGE and immunoblotting with antibodies against HA or GFP, PGK and porin. B. Ubp9, Ubp13 and Duf1 are found in fractions enriched in mitochondria. Fractions enriched in mitochondria were prepared from cells grown on lactate medium at 30°C and expressing chromosome-encoded Ubp9-GFP, Ubp13-HA (DB122-1D), or Duf1-HA (YDB107). Equal amounts of protein (80 µg) from the post-mitochondrial supernatant (PMS), crude mitochondria (mit1) and mitochondria further purified on a sucrose gradient (mit2) were loaded onto gels and analyzed by SDS-PAGE. Immunodetection was carried out with antibodies against porin, PGK, Sss1 and Vph1, as markers of the mitochondrial, cytosolic, ER and vacuolar compartments, respectively. Duf1-HA displayed some degradation products in mit1 fractions. C. Ubp9, Ubp13, and Duf1 are membrane-bound proteins. Fractions enriched in mitochondria (mit2) from cells producing HA-tagged Ubp9, Ubp13 or Duf1 (YDB105, YDB106 and YDB107) were sonicated on ice. Samples were left untreated (T) or subjected to ultracentrifugation at 100,000 g (S100, supernatant; P100, pellet) and then analyzed by SDS-PAGE and immunoblotting. Immunodetection was carried out with antibodies against HA, AAC and cytochrome b 2 (cyt b 2) as markers of the mitochondrial membrane and soluble fraction, respectively. (TIF) [file pone.0038071.s002.tif]

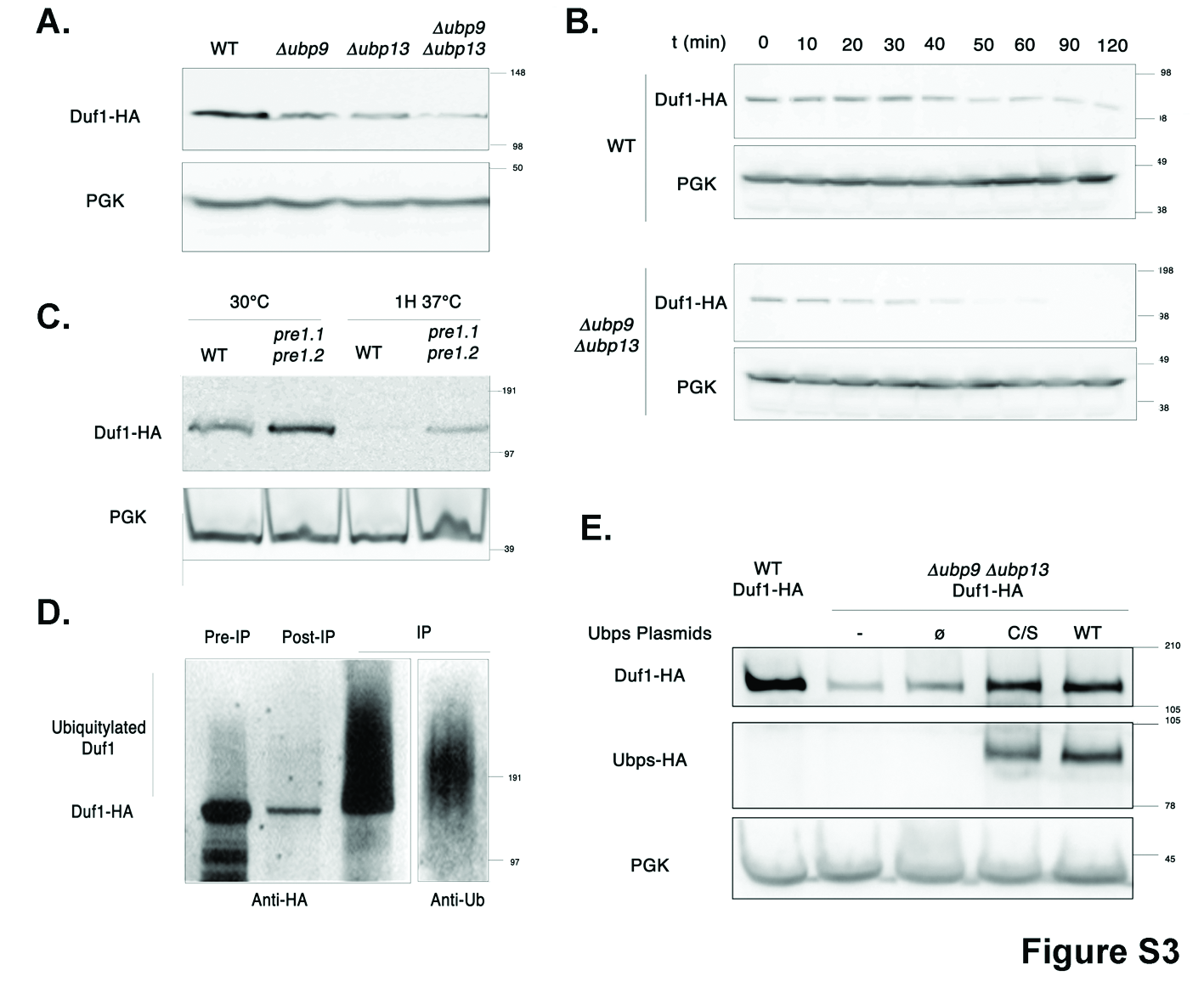

Supplement: Figure S3 — Duf1 is an unstable, ubiquitylated protein, further destabilized in the absence of its two protein partners, Ubp9 and Ubp13. A. Steady-state levels of Duf1 decrease in the Δubp9 Δubp13 double mutant. Crude extracts prepared from cells expressing chromosome-encoded Duf1-HA in wild-type, Δubp9, Δubp13 and Δubp9 Δubp13 backgrounds were grown on glucose-rich medium at 30°C and analyzed by western blotting with HA and PGK antibodies. B. The half-life of Duf1 is modified in the Δubp9 Δubp13 double mutant. Cells expressing Duf1-HA in wild-type or Δubp9 Δubp13 backgrounds were grown in glucose-rich medium at 30°C, and crude extracts were prepared at various times after the addition of cycloheximide (100 µg/ml). The stability of Duf1-HA was then monitored by SDS-PAGE and immunoblotting, with PGK antibody as a loading control. C. Duf1 is stabilized in the pre1-1 pre2-2 mutant cells. Crude extracts were prepared from cells expressing chromosome-encoded Duf1-HA gene, in wild-type or pre1-1 pre2-2 backgrounds, and growing exponentially in glucose-rich medium at 30°C, or after incubation for 1 h at 37°C. The steady-state level of Duf1-HA was then monitored by SDS-PAGE and immunoblotting, with anti-PGK antibody as a loading control. D. Duf1 is ubiquitylated. Cells growing exponentially on galactose-rich medium and producing chromosome-encoded Duf1-HA were subjected to immunoprecipitation in denaturing conditions with an anti-HA antibody. Input fractions (Pre), unbound material (Post) and immunoprecipitates (IP) were immunoblotted with the anti-HA and anti-ubiquitin antibodies. E. Duf1 stability depends on the physical presence of Ubp9 and Ubp13. Δubp9Δubp13 cells producing chromosome-encoded Duf1-HA either non transformed (-), or transformed with control empty plasmids (Ø), pFL38-UBP9C/S-HA plus pFL36-UBP13C/S-HA (C/S), or pFL38-UBP9-HA plus pFL38/pUL9-UBP13-HA (WT) were grown in glucose rich medium. Protein extracts prepared from wild-type cells expressing chromosome-encoded Duf1 [file pone.0038071.s003.tif]

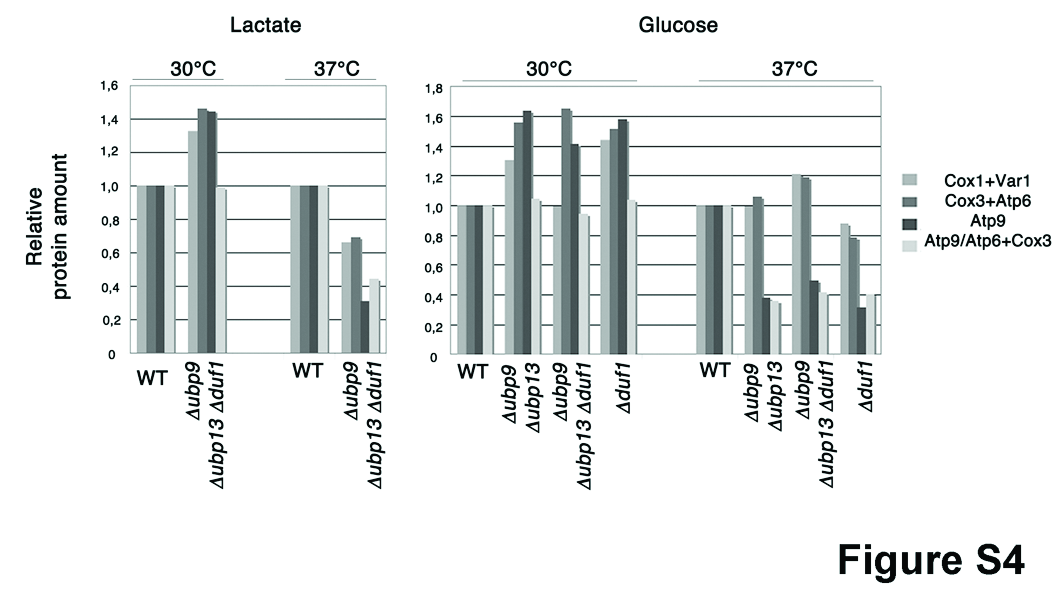

Supplement: Figure S4 — The deletion of UBP9 and UBP13 and the single deletion of DUF1 impair the synthesis of the mitochondrial ATP synthase subunit Atp9 at 37°C. The amount of each mitochondrial genome-encoded protein in mutant cells was determined relative to that in wild-type cells in the experiment described in Fig. 6. (TIF) [file pone.0038071.s004.tif]

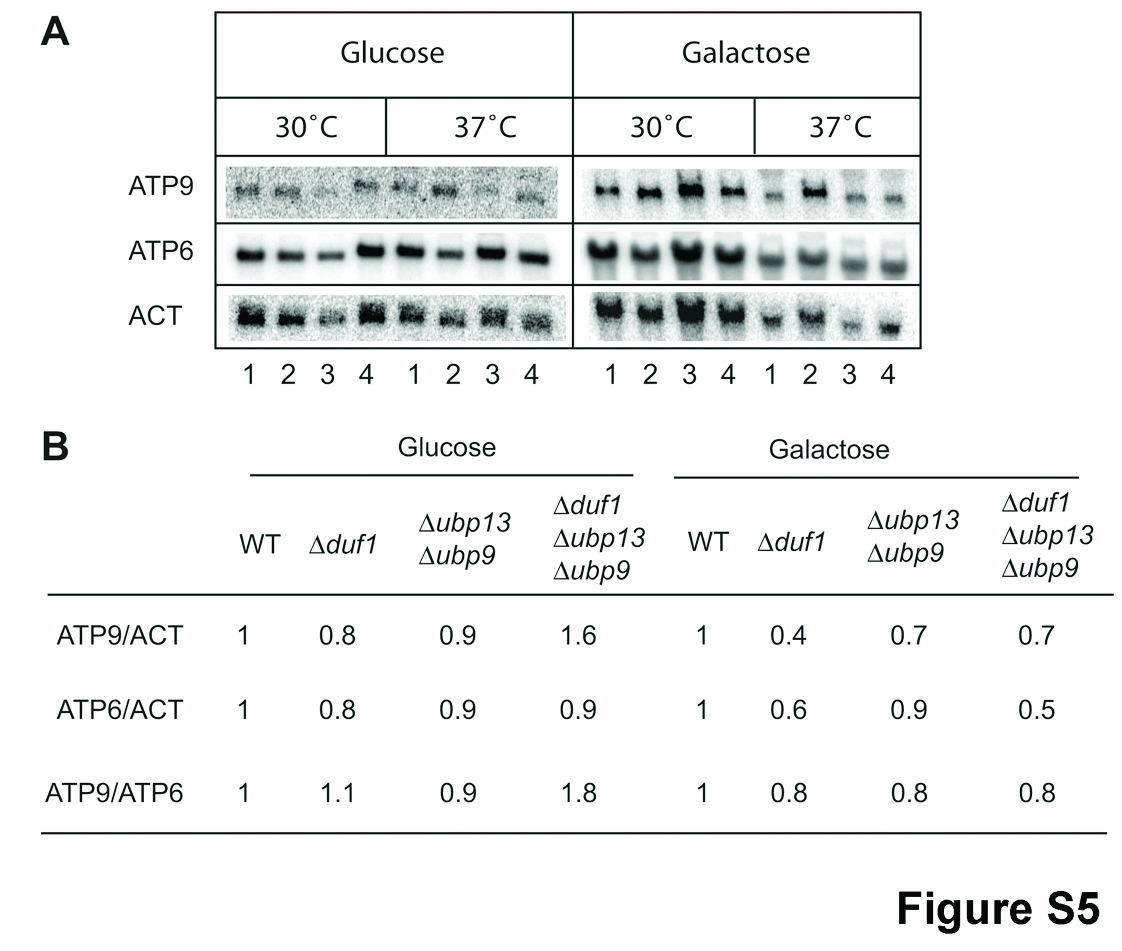

Supplement: Figure S5 — Northern analysis of yeast mRNAs. (A) Autoradiographs of washed filters for RNA extracted from yeast and separated in denaturing agarose gels are presented. Yeast strains were cultured with either glucose or galactose as the carbon source (as indicated above the autoradiographs) at two temperatures, 30°C and 37°C (as indicated above the autoradiographs). The samples are as follows: (1) Δduf1, (2) WT (wild type), (3) Δduf1, Δubp13, Δubp9, (4) Δubp13, Δubp9 (as indicated below the autoradiographs). The probes used for hybridization are indicated at the left of the autoradiographs: ACT, actin; ATP6 and ATP9. (B) Relative quantification results: the ratios of different hybridization signals are presented in the table. The 30°C/37°C ratios for the WT strain were taken for 1 in each series. (TIF) [file pone.0038071.s005.tif]

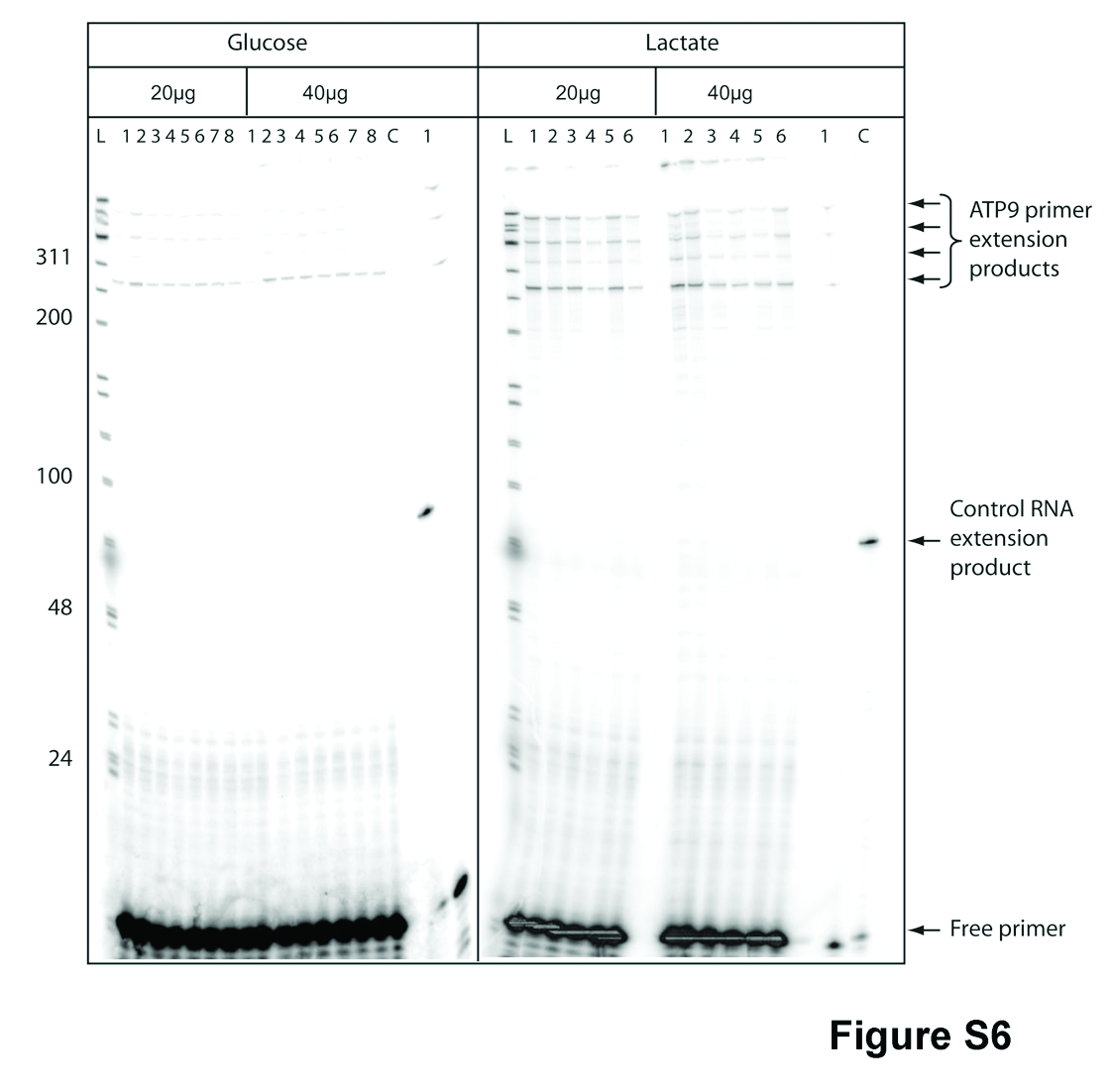

Supplement: Figure S6 — Analysis of ATP9 mRNA 5′-end maturation by primer extension. Autoradiographs of 10% polyacrylamide denaturing SDS-PAGE gels on which the products of primer extension were separated. Yeast strains were cultured in the presence of either glucose or lactate as the carbon source (as indicated above the lanes) at two temperatures, 30°C and 37°C. Two different amounts of yeast RNA were tested: 20 or 40 µg per assay (as indicated above the autoradiographs). The samples are as follows: (1) WT (wild type), 30°C; (2) WT, 37°C; (3) Δduf1, 30°C; (4) Δduf1, 37°C; (5) Δubp13 Δubp9, 30°C; (6) Δubp13 Δubp9, 37°C; (7) Δduf1 Δubp13 Δubp9, 30°C; (8) Δduf1 Δubp13 Δubp9, 37°C. “L” - labeled ladder from the primer extension kit (Promega). The size of a selection of fragments is indicated to the left of the panels. “C” the control extension assay obtained with RNA and the primer supplied in the kit (expected size: 84 nucleotides). On the right, the extension products obtained with yeast RNA or control RNAs are indicated by the arrows. (TIF) [file pone.0038071.s006.tif]
