# Supplementary material for: A Deubiquitylating Complex Required for Neosynthesis of a Yeast Mitochondrial ATP Synthase Subunit
Source: PLoS One. 2012 Jun 19;7(6):e38071. doi: 10.1371/journal.pone.0038071 (PMC3378586; doi:10.1371/journal.pone.0038071)
Supplement: Material S1 — Supplementary Materials and Methods. (DOC) [file pone.0038071.s007.doc]

**SUPPLEMENTAL DATA**

**Duf1 stability depends on the physical presence of its Ubps partners**

Given the interactions observed between Duf1 and Ubp9/Ubp13, we asked whether Duf1 is a substrate of these DUBs. We investigated the steady-state levels of Duf1-HA in ∆*ubp9* and *∆ubp13* single deletion mutants (Fig. S3A). In all cases, the steady-state level of Duf1 was clearly affected, and this effect was even more pronounced in the *∆ubp9 ∆ubp13* double mutant (Fig. S3A). This could result from a decrease in synthesis or enhanced degradation. We therefore assessed the stability of Duf1 in wild-type and *∆ubp9 ∆ubp13* cells after the inhibition of cytoplasmic protein synthesis with cycloheximide. The half-life of Duf1 was clearly shorter in ∆*ubp9* ∆*ubp13* cells than in wild-type cells (23 min versus 47 min) (Fig. S3B). This suggested that Duf1 stability *in vivo* depends on Ubp9 and Ubp13.

We investigated further the role of Ubp9/Ubp13 in Duf1 stability, by using catalytic null cysteine mutants of Ubp9 and Ubp13 with Cys to Ser mutation affecting the catalytic Cys in the Cys box of each Ubp . *∆ubp9 ∆ubp13* cells producing chromosome-encoded Duf1-HA were cotransformed with centromeric plasmids carrying a wild-type or mutated version of *UBP9* and *UBP13* (Fig. S3E). The steady-state levels of Duf1 clearly increased following the production of wild-type or catalytically inactive versions of Ubp9 and Ubp13. This indicates that Duf1 is likely not a substrate of Ubp9 and Ubp13. Therefore, like other components of many multiprotein complexes which are destabilized in the absence of their partners, the stability of Duf1 seems to depend on the physical presence of Ubp9 and Ubp13, whether active or not. The steady-state levels of Ubp9 and Ubp13, however, were not reproducibly modified in *∆duf1* mutants (data not shown).

To address whether Duf1 is degraded by the ubiquitin-proteasome pathway, we generated a tagged version of Duf1 in the thermosensitive *pre1-1 pre2-2* double mutant, which is deficient in proteasome catalytic activity , and in the corresponding wild-type cells. Duf1 was stabilized in the *pre1-1 pre2-2* background, being 1.6 times as stable as in wild-type cells at 30°C and twice as stable at 37°C (Fig. S3C). These observations suggest that Duf1 may be ubiquitylated in wild-type cells. Indeed, the immunoprecipitation of Duf1-HA with an anti-HA antibody in denaturing conditions, followed by detection with an anti-ubiquitin antibody, resulted in the detection of ubiquitylated Duf1 species (Fig. S3D). Therefore, Duf1 appears to be an unstable ubiquitylated protein, the half-life of which depends on proteasome activity and on the physical presence but not the catalytic activity of Ubp9 or Ubp13.

**SUPPLEMENTAL MATERIALS AND METHODS**

**Subcellular fractionation**

For subcellular fractionation, cells grown on galactose were harvested, resuspended in spheroplast buffer (1.2 M sorbitol 20 mM KPi, pH 7.4) and spheroplasts were generated by adding 0.2 mg/ml zymolyase 20T (ICN Biomedicals). Spheroplasts were lysed in ice-cold lysis buffer (50 mM Tris-HCl, pH 7.5, 1 mM EDTA 0.2 M sorbitol, 1 mM PMSF plus a mixture of protease inhibitors (Complete from Roche Diagnostics, used in all the experiments)). The suspension was homogenized on ice, by 20 strokes with a Dounce homogenizer. Homogenates were centrifuged at 3000g to remove cell debris and the resulting total extract was centrifuged at 13,000 g to obtain pellets (P13) and supernatant fractions (S13). Proteins from S13 were precipitated with 10% TCA. P13 were directly resuspended in SDS sample buffer.

For subcellular fractionation of spheroplastes, cells were treated as described above, except that homogenates were centrifuged at 100 g for elimination of cell debris. Fractions enriched in mitochondria were purified from protoplasts prepared from yeast cells grown in galactose or lactate medium, as previously described . Briefly, the crude mitochondrial fraction (12,000g pellet, mit1) was resuspended and loaded on a discontinuous sucrose gradient (15, 23, 32, and 60% sucrose), centrifuged for 60 minutes at 135,000 g at 4°C in an SW41 rotor (Beckman). The fraction enriched in mitochondria (mit2) was recovered from the 60%/32% interface . The protein concentration of crude mitochondrial fractions (mit1), purified mitochondrial fractions (mit2) and post-mitochondrial supernatants (PMS) was determined with Bradford’s reagent (Sigma) or the Coomassie protein assay reagent (Pierce). Protein samples (80 ∆g) were analyzed by SDS-PAGE and electroblotting onto PVDF or nitrocellulose membranes. The fractions enriched in mitochondria (mit2) were sonicated on ice (Bioblock Scientific, 3 x 30 s, duty cycle 40%, output 5) and the soluble fraction was separated from the membrane fraction by centrifugation at 100,000 x g for 1 h at 4°C. Total (T), pellet (P) and supernatant (S) fractions were then analyzed by SDS-PAGE using antibodies against HA, AAC and cytochrome b_2_ (cyt b_2_) as markers of the mitochondrial membrane and soluble fraction, respectively.

**Northern blots analysis**

Total yeast RNA was isolated by Trizol (Invitrogen) treatment, as described by the manufacturer, following cell disruption with glass beads (0.35mm), separated by electrophoresis in denaturating formaldehyde agarose gels, as previously described . [2,3]. RNA was then blotted onto Hybond-N nylon membranes (GE Healthcare), which were hybridized with the following 5'-end ^32^P-labeled oligonucleotide probes:

ATP9: GCTCCTAATAAACCAATTGTTGAGA;

ATP6: TTACCTCCAATTTGTCCTTTAAGC,

COX2: ACAGCTGGAAAAATTGTTCAAATA,

Actin: ACCAGAACCGTTATCAATAACCAA.

The washed blots were analyzed with a Typhoon-Trio phosphor imager and ImageQuant software.

**Primer extension**

The oligonucleotide complementary to the mRNA of *ATP9* (GCTCCTAATAAACCAATTGTTGAGA, coordinates 46723-46753 according to the yeast mitochondrial DNA sequence was ^32^P-labeled by T4 polynucleotide kinase and used for extension with AMV-reverse transcriptase on total yeast RNA or the control RNA, with the primer extension kit (Promega), as described by the manufacturer. The extension products were separated by electrophoresis in 40 cm long 10% polyacrylamide denaturing urea gels and analyzed with a Typhoon-Trio phosphor imager and ImageQuant software.

***References****:*

*1. Meisinger C, Pfanner N, Truscott KN (2006) Isolation of yeast mitochondria.Methods Mol Biol 313: 33-39.*

*2. Lehrach H, Diamond D, Wozney JM, Boedtker H (1977) RNA molecular weight determinations by gel electrophoresis under denaturing conditions, a critical reexamination. Biochemistry 16: 4743-4751.*

*3. Sambrook J, Fritsh E, Maniatis T (1982) Molecular Cloning: a Laboratory Manual; Press CSHL, editor. New York.*

*4. Foury F, Roganti T, Lecrenier N, Purnelle B (1998) The complete sequence of the mitochondrial genome of Saccharomyces cerevisiae. FEBS Lett 440: 325-331*
